# Supplementary material for: Birth and death of links control disease spreading in empirical contact networks
Source: Sci Rep. 2014 May 23;4:4999. doi: 10.1038/srep04999 (PMC4031628; doi:10.1038/srep04999)
Supplement: Supplementary Information [file srep04999-s1.pdf]

Birth and death of links control disease spreading in empirical contact networks

Petter Holme[1,2,3,4]

Fredrik Liljeros[3,4]

[1]Department of Energy Science, Sungkyunkwan University, 440-746 Suwon, Korea

[2]IceLab, Department of Physics, Umeå University, 90187 Umeå, Sweden

[3]Department of Sociology, Stockholm University, 10961 Stockholm, Sweden

[4]Institute for Futures Study, 10131 Stockholm, Sweden

E-mail address: holme@skku.edu

Supplementary Information Legend

SI1 These figures shows plots similar to Fig. 4 of the paper for all the datasets we use. It shows the average outbreak size (the fraction of individuals once infected) as a function of the two parameter values of the SIR model (the per-contact transmission probability and the duration of the infection). *SI2* Here we show the maximum positive $\Delta_{pos}$and negative Δ_{neg} deviations between the empirical and reference data. The bottom of the bar shows the maximum negative deviation and the top of the bar shows the maximum positive deviation. *SI3* This figure shows exactly the same thing as SI1 but for the SIS model. *SI4* These plots are similar to SI1 and SI3 but they show, for the SIS model, the average number of infection events per individual. So instead of counting the persons that at some point has been infected, here we also include reinfections (which are impossible in the SIR model). *SI5* This plot is identical to SI2, but for the SIS model.

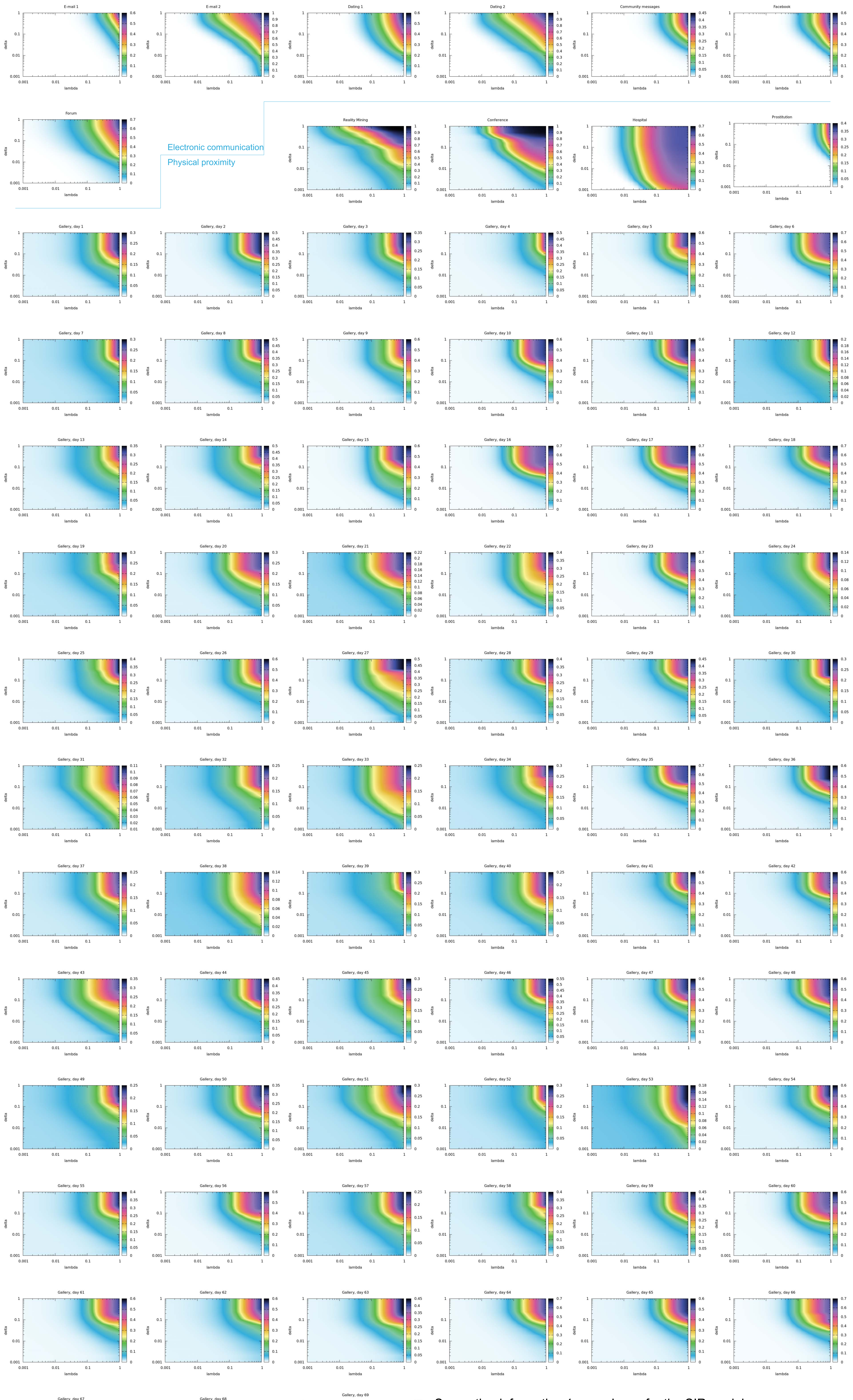

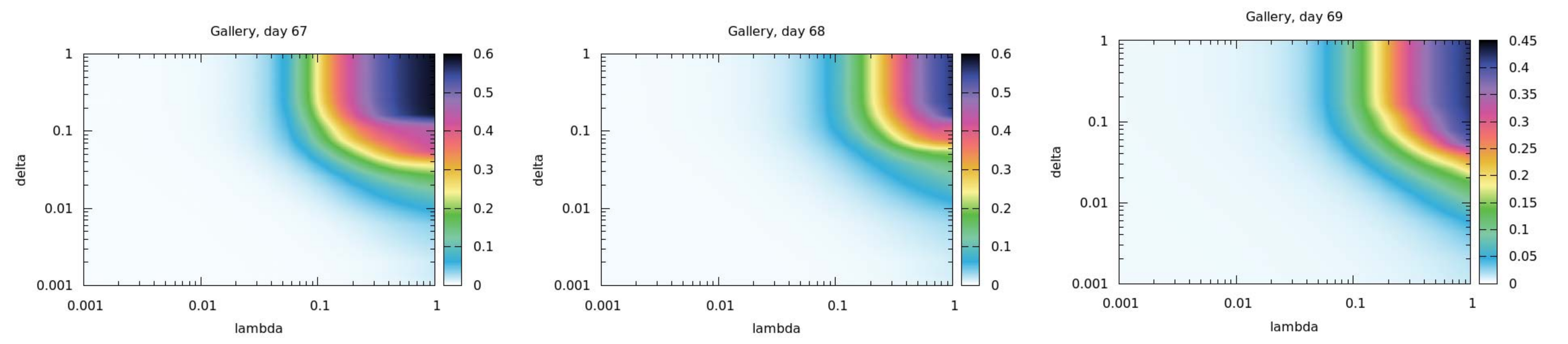

Supporting information 1, prevalence for the SIR model

Petter Holme, Fredrik Liljeros

Beginning and end times of relationships control disease spreading in empirical contact networks

Original

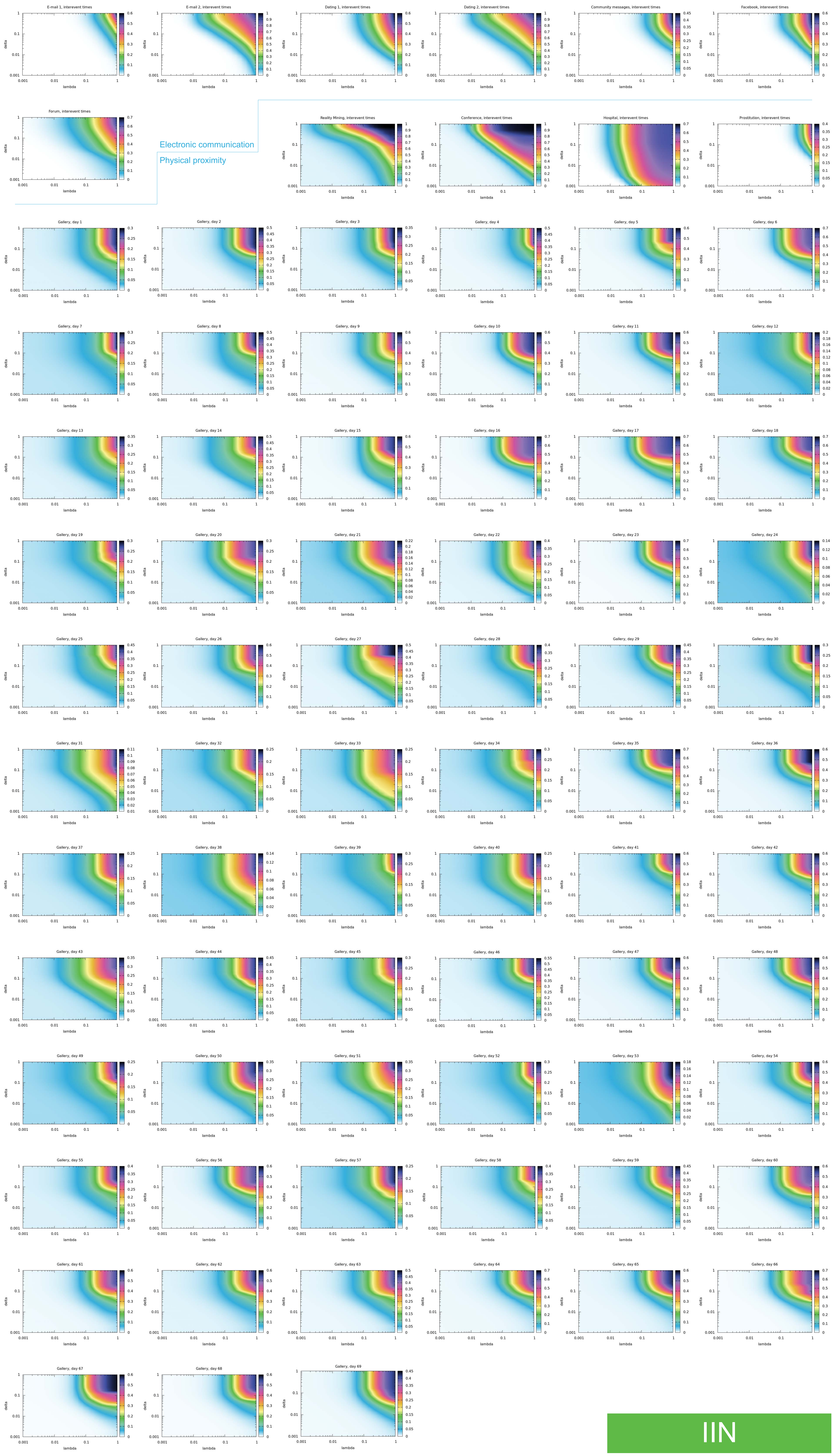
E-mail 1, interevent times
E-mail 2, interevent times
Dating 1, interevent times
Dating 2, interevent times
Community messages, interevent times
Facebook, interevent times
Forum, interevent times
Electronic communication
Physical proximity
Reality Mining, interevent times
Conference, interevent times
Hospital, interevent times
Prostitution, interevent times
Gallery, day 1
Gallery, day 2
Gallery, day 3
Gallery, day 4
Gallery, day 5
Gallery, day 6
Gallery, day 7
Gallery, day 8
Gallery, day 9
Gallery, day 10
Gallery, day 11
Gallery, day 12
Gallery, day 13
Gallery, day 14
Gallery, day 15
Gallery, day 16
Gallery, day 17
Gallery, day 18
Gallery, day 19
Gallery, day 20
Gallery, day 21
Gallery, day 22
Gallery, day 23
Gallery, day 24
Gallery, day 25
Gallery, day 26
Gallery, day 27
Gallery, day 28
Gallery, day 29
Gallery, day 30
Gallery, day 31
Gallery, day 32
Gallery, day 33
Gallery, day 34
Gallery, day 35
Gallery, day 36
Gallery, day 37
Gallery, day 38
Gallery, day 39
Gallery, day 40
Gallery, day 41
Gallery, day 42
Gallery, day 43
Gallery, day 44
Gallery, day 45
Gallery, day 46
Gallery, day 47
Gallery, day 48
Gallery, day 49
Gallery, day 50
Gallery, day 51
Gallery, day 52
Gallery, day 53
Gallery, day 54
Gallery, day 55
Gallery, day 56
Gallery, day 57
Gallery, day 58
Gallery, day 59
Gallery, day 60
Gallery, day 61
Gallery, day 62
Gallery, day 63
Gallery, day 64
Gallery, day 65
Gallery, day 66
Gallery, day 67
Gallery, day 68
Gallery, day 69
delta
lambda

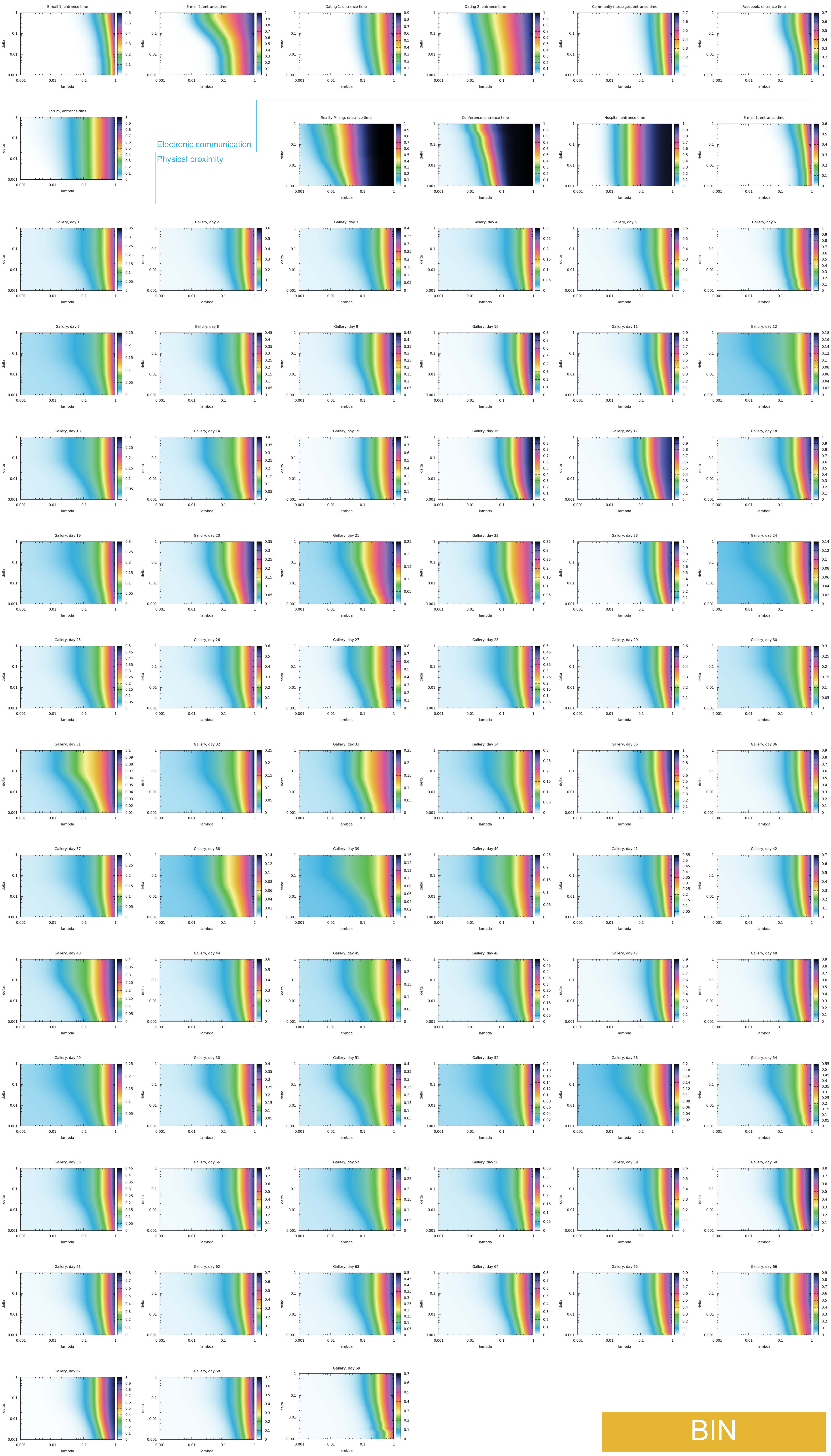
E-mail 1, entrance time
E-mail 2, entrance time
Dating 1, entrance time
Dating 2, entrance time
Community messages, entrance time
Facebook, entrance time
Forum, entrance time
Electronic communication
Physical proximity
Reality Mining, entrance time
Conference, entrance time
Hospital, entrance time
E-mail 1, entrance time
Gallery, day 1
Gallery, day 2
Gallery, day 3
Gallery, day 4
Gallery, day 5
Gallery, day 6
Gallery, day 7
Gallery, day 8
Gallery, day 9
Gallery, day 10
Gallery, day 11
Gallery, day 12
Gallery, day 13
Gallery, day 14
Gallery, day 15
Gallery, day 16
Gallery, day 17
Gallery, day 18
Gallery, day 19
Gallery, day 20
Gallery, day 21
Gallery, day 22
Gallery, day 23
Gallery, day 24
Gallery, day 25
Gallery, day 26
Gallery, day 27
Gallery, day 28
Gallery, day 29
Gallery, day 30
Gallery, day 31
Gallery, day 32
Gallery, day 33
Gallery, day 34
Gallery, day 35
Gallery, day 36
Gallery, day 37
Gallery, day 38
Gallery, day 39
Gallery, day 40
Gallery, day 41
Gallery, day 42
Gallery, day 43
Gallery, day 44
Gallery, day 45
Gallery, day 46
Gallery, day 47
Gallery, day 48
Gallery, day 49
Gallery, day 50
Gallery, day 51
Gallery, day 52
Gallery, day 53
Gallery, day 54
Gallery, day 55
Gallery, day 56
Gallery, day 57
Gallery, day 58
Gallery, day 59
Gallery, day 60
Gallery, day 61
Gallery, day 62
Gallery, day 63
Gallery, day 64
Gallery, day 65
Gallery, day 66
Gallery, day 67
Gallery, day 68
Gallery, day 69
delta
lambda
BIN

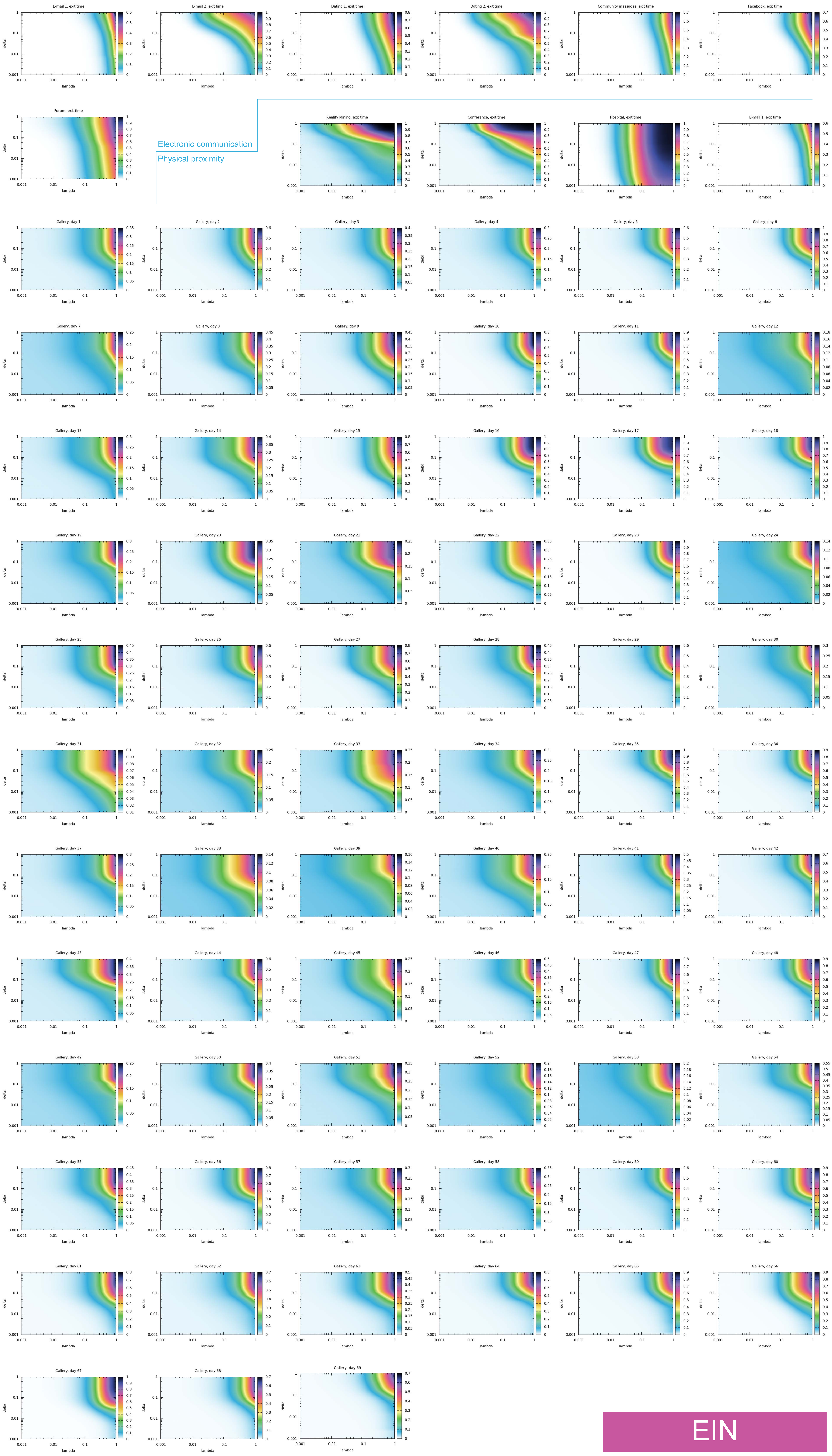
E-mail 1, exit time
E-mail 2, exit time
Dating 1, exit time
Dating 2, exit time
Community messages, exit time
Facebook, exit time
Forum, exit time
Electronic communication
Physical proximity
Reality Mining, exit time
Conference, exit time
Hospital, exit time
E-mail 1, exit time
Gallery, day 1
Gallery, day 2
Gallery, day 3
Gallery, day 4
Gallery, day 5
Gallery, day 6
Gallery, day 7
Gallery, day 8
Gallery, day 9
Gallery, day 10
Gallery, day 11
Gallery, day 12
Gallery, day 13
Gallery, day 14
Gallery, day 15
Gallery, day 16
Gallery, day 17
Gallery, day 18
Gallery, day 19
Gallery, day 20
Gallery, day 21
Gallery, day 22
Gallery, day 23
Gallery, day 24
Gallery, day 25
Gallery, day 26
Gallery, day 27
Gallery, day 28
Gallery, day 29
Gallery, day 30
Gallery, day 31
Gallery, day 32
Gallery, day 33
Gallery, day 34
Gallery, day 35
Gallery, day 36
Gallery, day 37
Gallery, day 38
Gallery, day 39
Gallery, day 40
Gallery, day 41
Gallery, day 42
Gallery, day 43
Gallery, day 44
Gallery, day 45
Gallery, day 46
Gallery, day 47
Gallery, day 48
Gallery, day 49
Gallery, day 50
Gallery, day 51
Gallery, day 52
Gallery, day 53
Gallery, day 54
Gallery, day 55
Gallery, day 56
Gallery, day 57
Gallery, day 58
Gallery, day 59
Gallery, day 60
Gallery, day 61
Gallery, day 62
Gallery, day 63
Gallery, day 64
Gallery, day 65
Gallery, day 66
Gallery, day 67
Gallery, day 68
Gallery, day 69
EIN

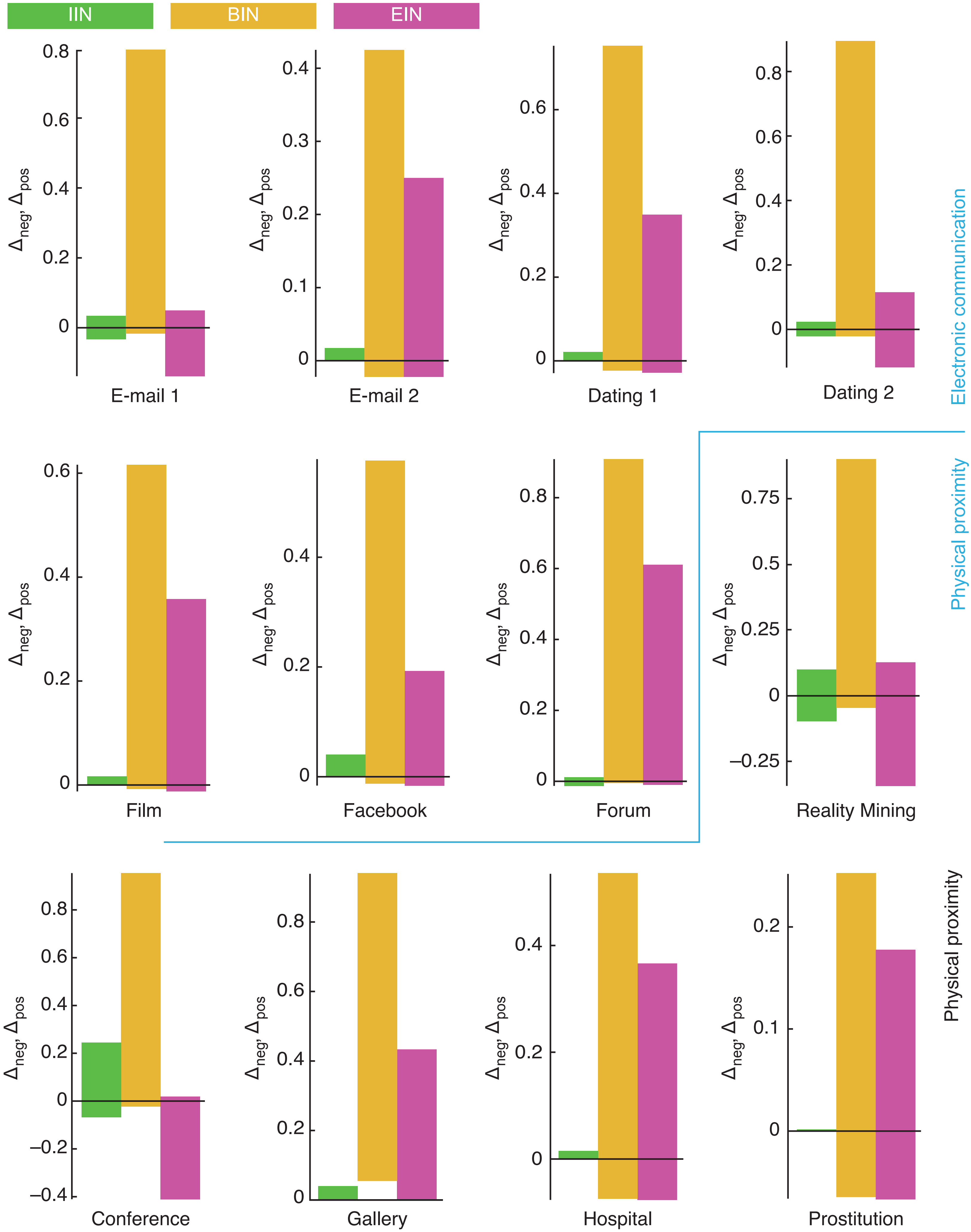

Supporting information 2, maximum deviations for the SIR model

Petter Holme, Fredrik Liljeros

Beginning and end times of relationships control disease spreading in empirical contact networks

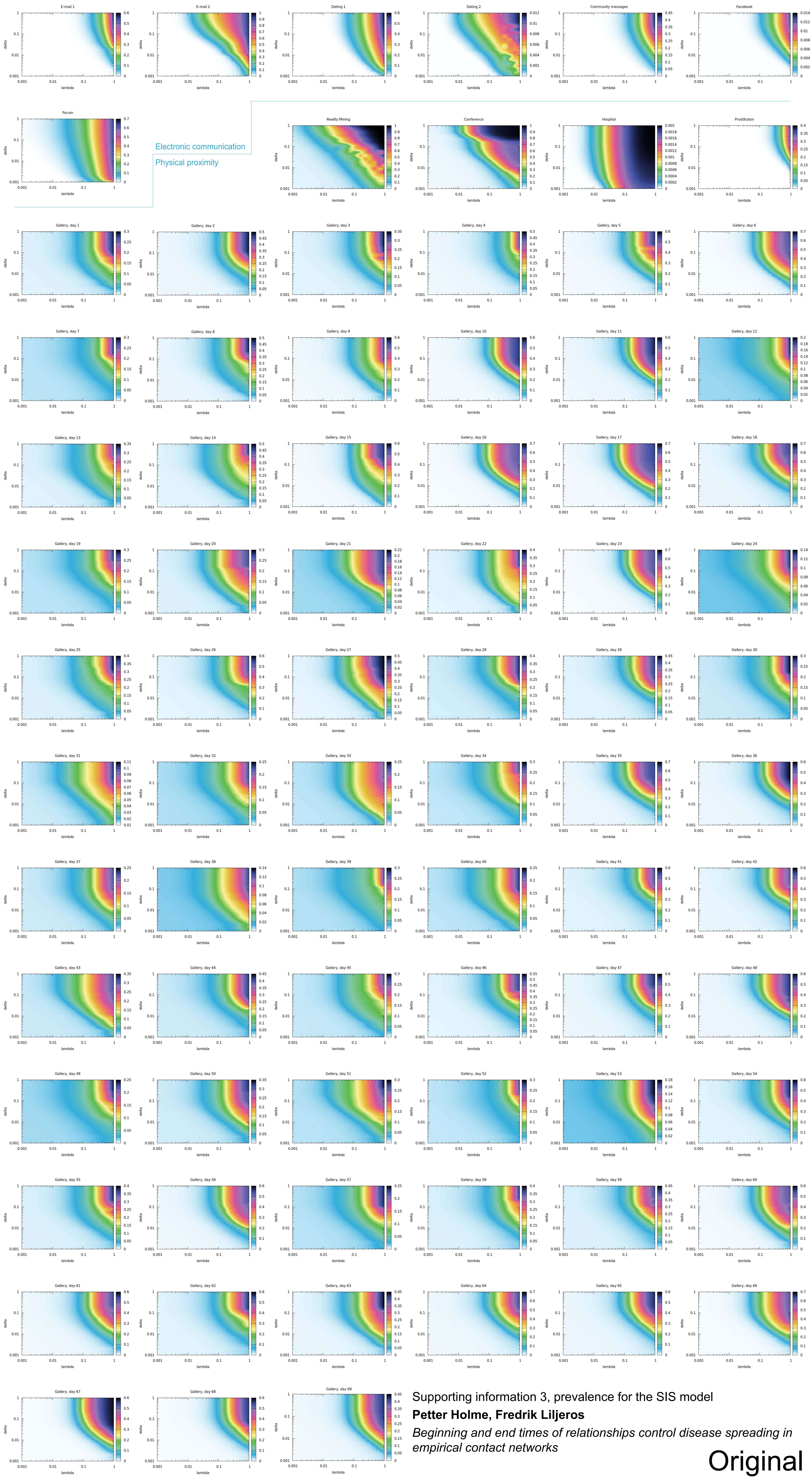

Supporting information 3, prevalence for the SIS model

Petter Holme, Fredrik Liljeros

Beginning and end times of relationships control disease spreading in empirical contact networks

Original

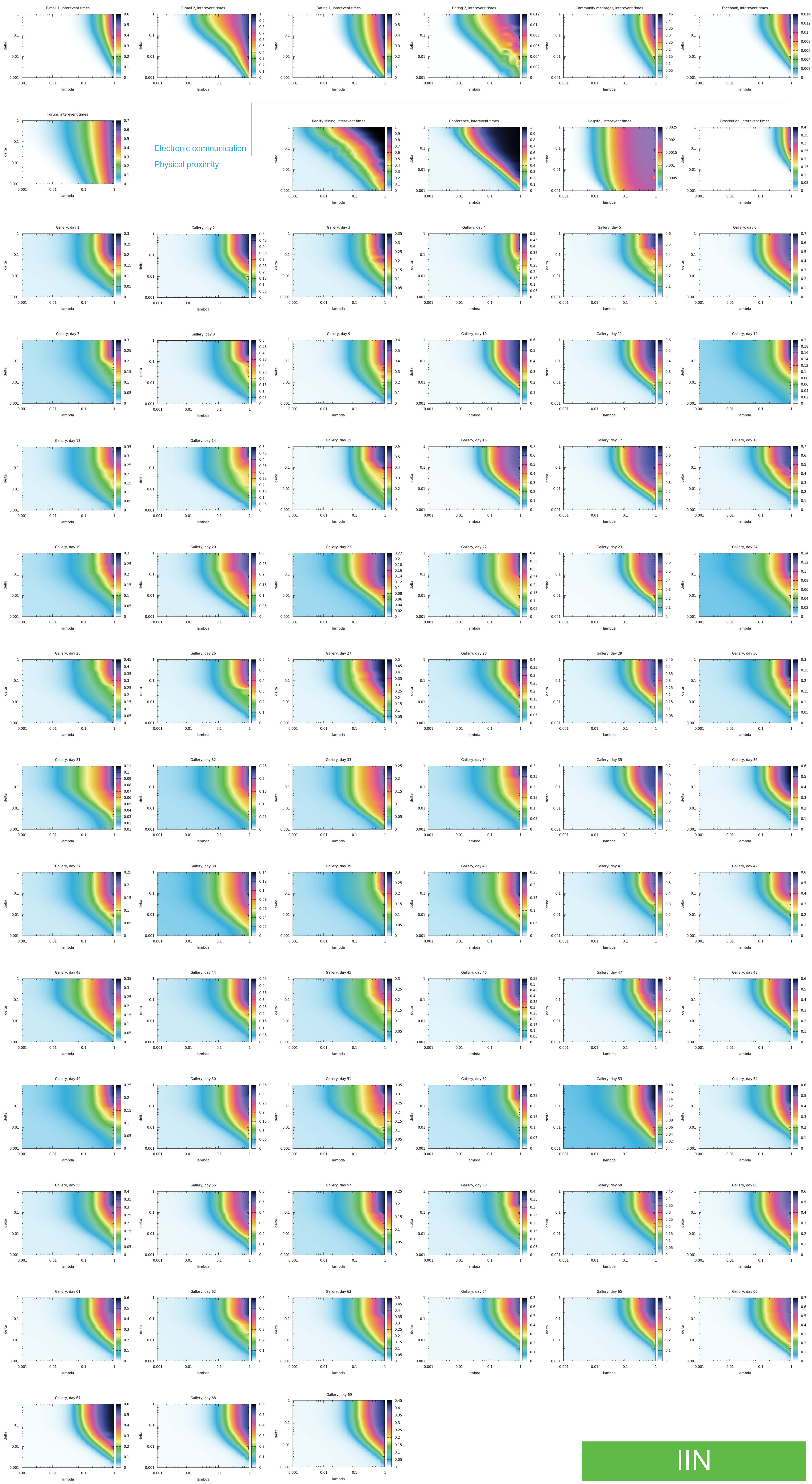
E-mail 1, interevent times
E-mail 2, interevent times
Dating 1, interevent times
Dating 2, interevent times
Community messages, interevent times
Facebook, interevent times
Forum, interevent times
Electronic communication
Physical proximity
Reality Mining, interevent times
Conference, interevent times
Hospital, interevent times
Prostitution, interevent times
Gallery, day 1
Gallery, day 2
Gallery, day 3
Gallery, day 4
Gallery, day 5
Gallery, day 6
Gallery, day 7
Gallery, day 8
Gallery, day 9
Gallery, day 10
Gallery, day 11
Gallery, day 12
Gallery, day 13
Gallery, day 14
Gallery, day 15
Gallery, day 16
Gallery, day 17
Gallery, day 18
Gallery, day 19
Gallery, day 20
Gallery, day 21
Gallery, day 22
Gallery, day 23
Gallery, day 24
Gallery, day 25
Gallery, day 26
Gallery, day 27
Gallery, day 28
Gallery, day 29
Gallery, day 30
Gallery, day 31
Gallery, day 32
Gallery, day 33
Gallery, day 34
Gallery, day 35
Gallery, day 36
Gallery, day 37
Gallery, day 38
Gallery, day 39
Gallery, day 40
Gallery, day 41
Gallery, day 42
Gallery, day 43
Gallery, day 44
Gallery, day 45
Gallery, day 46
Gallery, day 47
Gallery, day 48
Gallery, day 49
Gallery, day 50
Gallery, day 51
Gallery, day 52
Gallery, day 53
Gallery, day 54
Gallery, day 55
Gallery, day 56
Gallery, day 57
Gallery, day 58
Gallery, day 59
Gallery, day 60
Gallery, day 61
Gallery, day 62
Gallery, day 63
Gallery, day 64
Gallery, day 65
Gallery, day 66
Gallery, day 67
Gallery, day 68
Gallery, day 69
lambda
delta

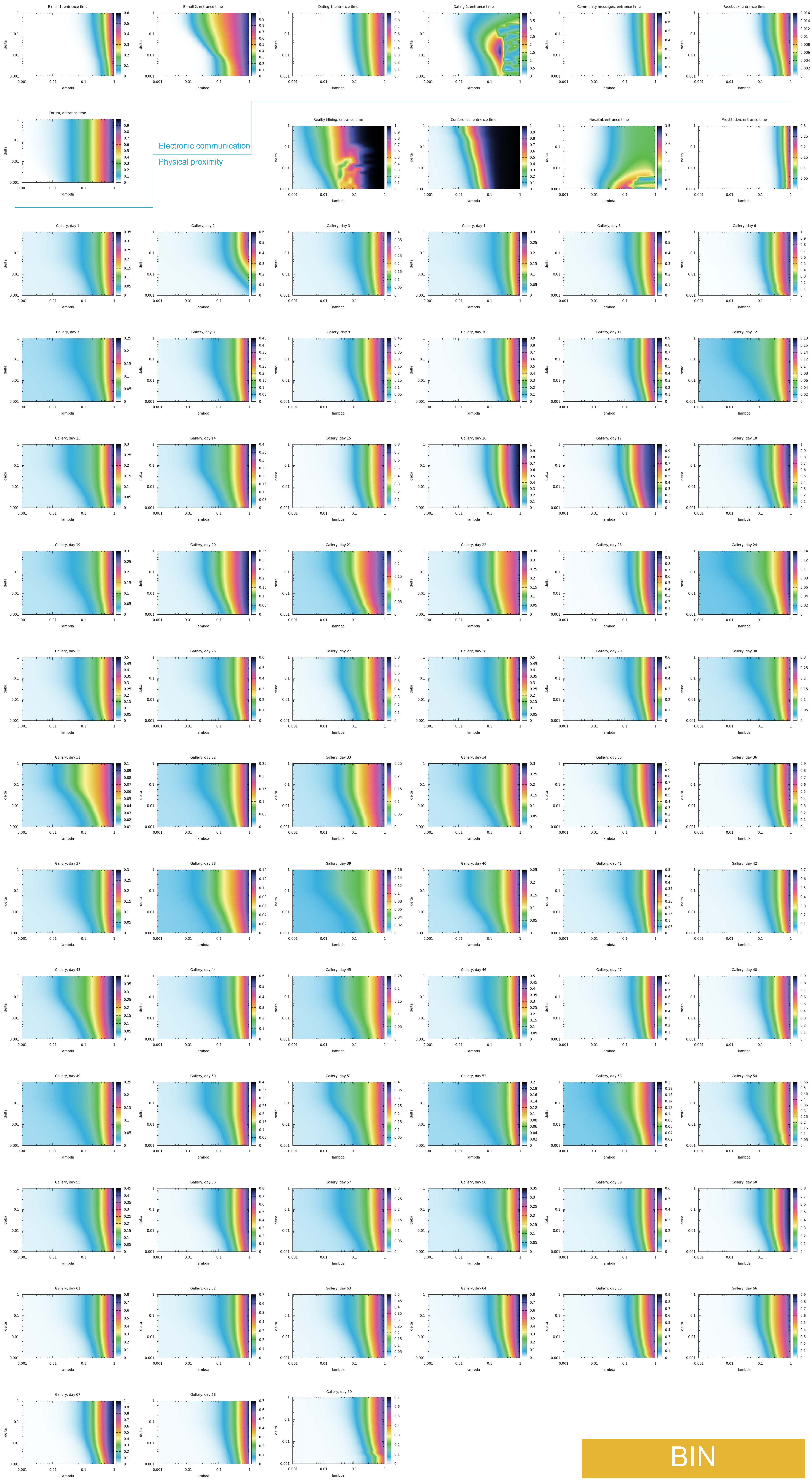
E-mail 1, entrance time
E-mail 2, entrance time
Dating 1, entrance time
Dating 2, entrance time
Community messages, entrance time
Facebook, entrance time
Forum, entrance time
Electronic communication
Physical proximity
Reality Mining, entrance time
Conference, entrance time
Hospital, entrance time
Prostitution, entrance time
Gallery, day 1
Gallery, day 2
Gallery, day 3
Gallery, day 4
Gallery, day 5
Gallery, day 6
Gallery, day 7
Gallery, day 8
Gallery, day 9
Gallery, day 10
Gallery, day 11
Gallery, day 12
Gallery, day 13
Gallery, day 14
Gallery, day 15
Gallery, day 16
Gallery, day 17
Gallery, day 18
Gallery, day 19
Gallery, day 20
Gallery, day 21
Gallery, day 22
Gallery, day 23
Gallery, day 24
Gallery, day 25
Gallery, day 26
Gallery, day 27
Gallery, day 28
Gallery, day 29
Gallery, day 30
Gallery, day 31
Gallery, day 32
Gallery, day 33
Gallery, day 34
Gallery, day 35
Gallery, day 36
Gallery, day 37
Gallery, day 38
Gallery, day 39
Gallery, day 40
Gallery, day 41
Gallery, day 42
Gallery, day 43
Gallery, day 44
Gallery, day 45
Gallery, day 46
Gallery, day 47
Gallery, day 48
Gallery, day 49
Gallery, day 50
Gallery, day 51
Gallery, day 52
Gallery, day 53
Gallery, day 54
Gallery, day 55
Gallery, day 56
Gallery, day 57
Gallery, day 58
Gallery, day 59
Gallery, day 60
Gallery, day 61
Gallery, day 62
Gallery, day 63
Gallery, day 64
Gallery, day 65
Gallery, day 66
Gallery, day 67
Gallery, day 68
Gallery, day 69
delta
lambda
BIN

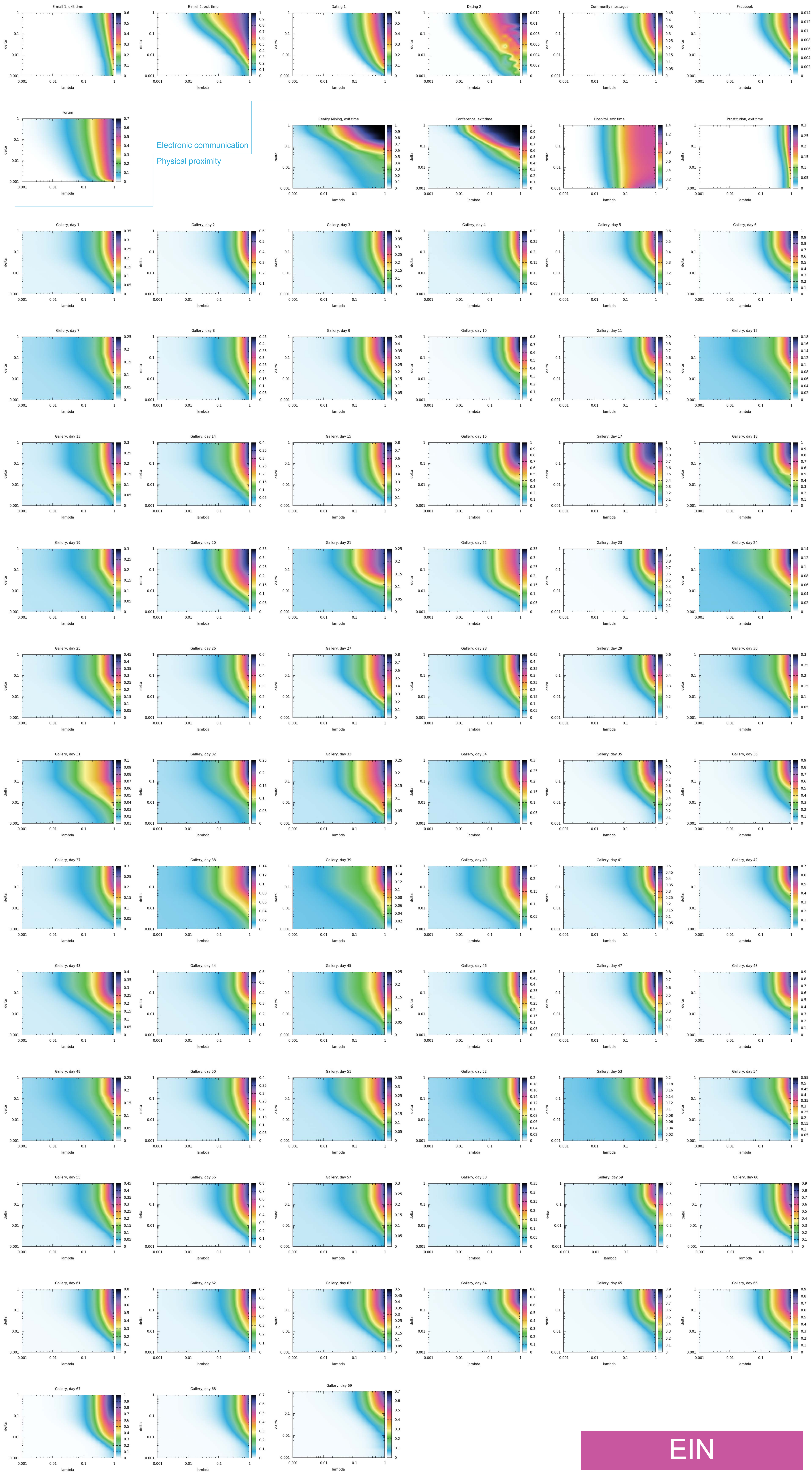

E-mail 1, exit time
E-mail 2, exit time
Dating 1
Dating 2
Community messages
Facebook
Forum
Electronic communication
Physical proximity
Reality Mining, exit time
Conference, exit time
Hospital, exit time
Prostitution, exit time
Gallery, day 1
Gallery, day 2
Gallery, day 3
Gallery, day 4
Gallery, day 5
Gallery, day 6
Gallery, day 7
Gallery, day 8
Gallery, day 9
Gallery, day 10
Gallery, day 11
Gallery, day 12
Gallery, day 13
Gallery, day 14
Gallery, day 15
Gallery, day 16
Gallery, day 17
Gallery, day 18
Gallery, day 19
Gallery, day 20
Gallery, day 21
Gallery, day 22
Gallery, day 23
Gallery, day 24
Gallery, day 25
Gallery, day 26
Gallery, day 27
Gallery, day 28
Gallery, day 29
Gallery, day 30
Gallery, day 31
Gallery, day 32
Gallery, day 33
Gallery, day 34
Gallery, day 35
Gallery, day 36
Gallery, day 37
Gallery, day 38
Gallery, day 39
Gallery, day 40
Gallery, day 41
Gallery, day 42
Gallery, day 43
Gallery, day 44
Gallery, day 45
Gallery, day 46
Gallery, day 47
Gallery, day 48
Gallery, day 49
Gallery, day 50
Gallery, day 51
Gallery, day 52
Gallery, day 53
Gallery, day 54
Gallery, day 55
Gallery, day 56
Gallery, day 57
Gallery, day 58
Gallery, day 59
Gallery, day 60
Gallery, day 61
Gallery, day 62
Gallery, day 63
Gallery, day 64
Gallery, day 65
Gallery, day 66
Gallery, day 67
Gallery, day 68
Gallery, day 69
lambda
delta
EIN

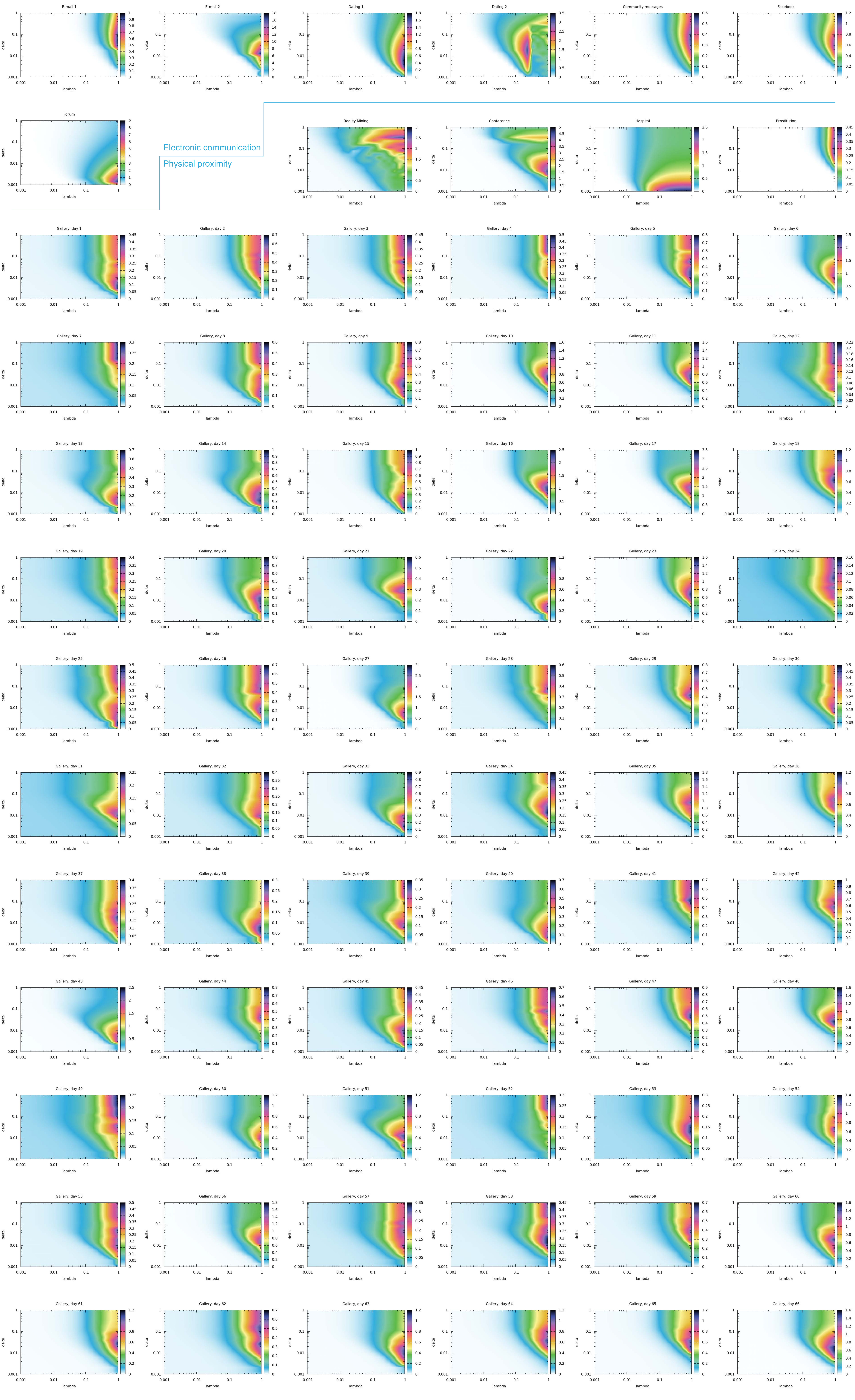

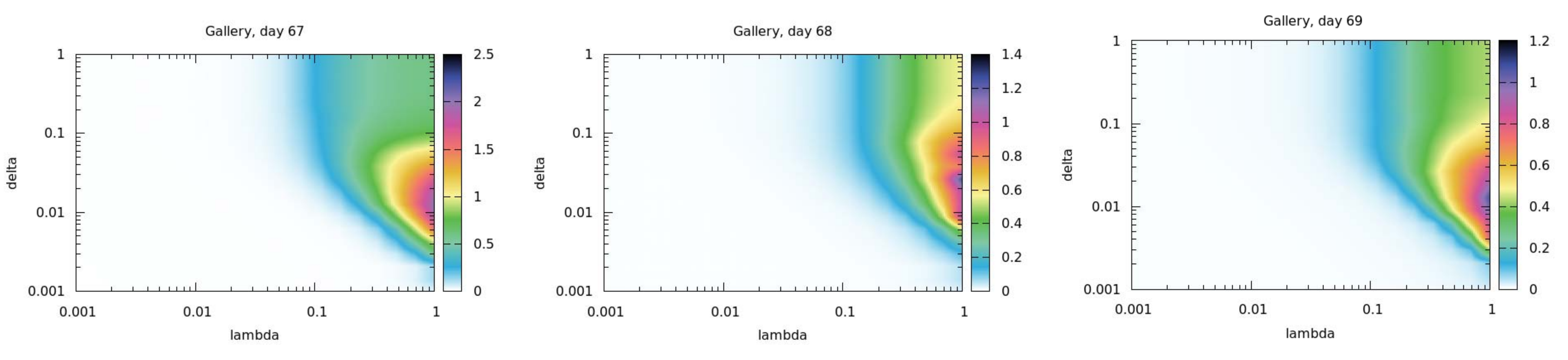

Supporting information 4, number of infections for the SIS model

Petter Holme, Fredrik Liljeros

Beginning and end times of relationships control disease spreading in empirical contact networks

Original

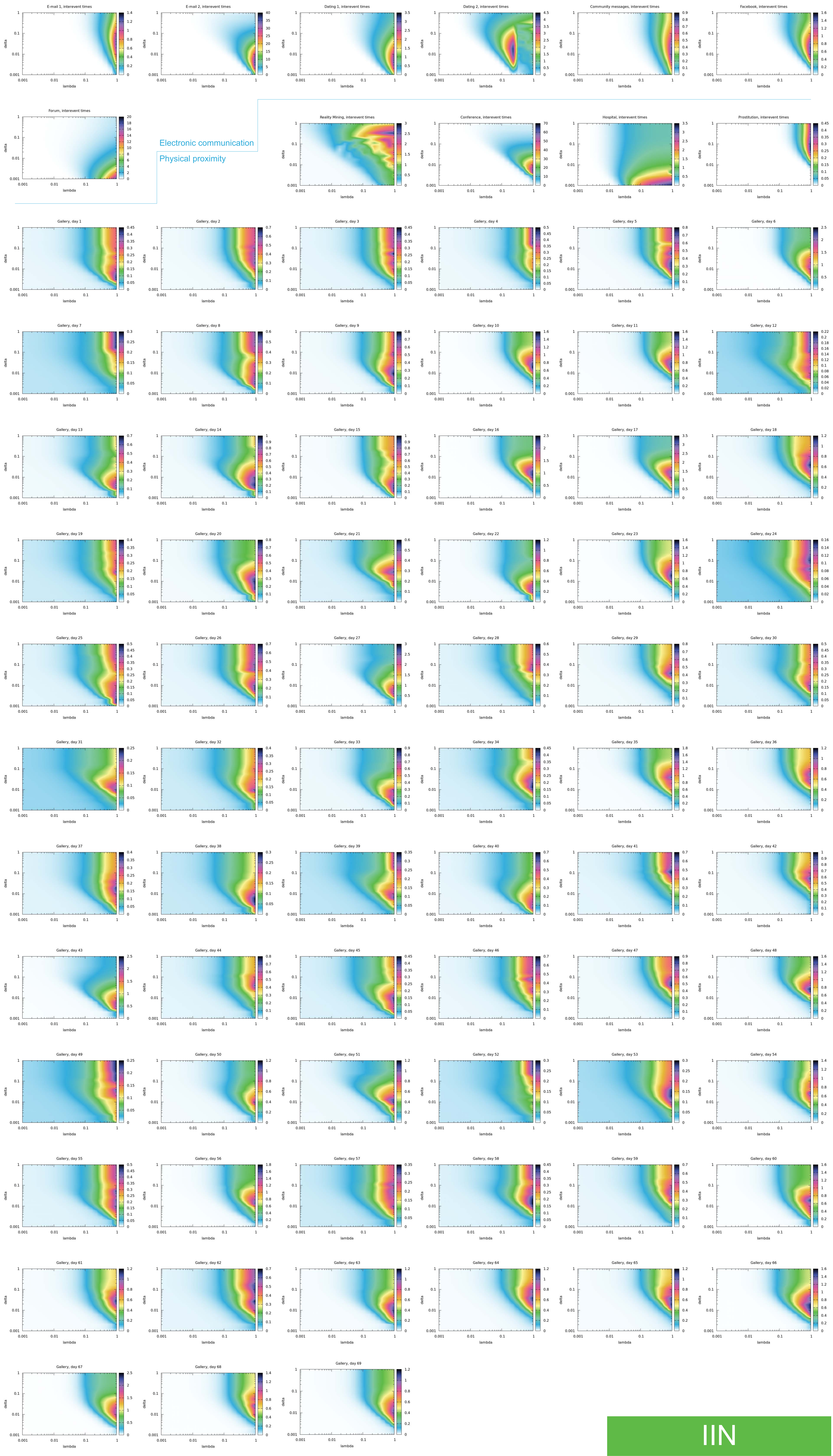
E-mail 1, interevent times
E-mail 2, interevent times
Dating 1, interevent times
Dating 2, interevent times
Community messages, interevent times
Facebook, interevent times
Forum, interevent times
Electronic communication
Physical proximity
Reality Mining, interevent times
Conference, interevent times
Hospital, interevent times
Prostitution, interevent times
Gallery, day 1
Gallery, day 2
Gallery, day 3
Gallery, day 4
Gallery, day 5
Gallery, day 6
Gallery, day 7
Gallery, day 8
Gallery, day 9
Gallery, day 10
Gallery, day 11
Gallery, day 12
Gallery, day 13
Gallery, day 14
Gallery, day 15
Gallery, day 16
Gallery, day 17
Gallery, day 18
Gallery, day 19
Gallery, day 20
Gallery, day 21
Gallery, day 22
Gallery, day 23
Gallery, day 24
Gallery, day 25
Gallery, day 26
Gallery, day 27
Gallery, day 28
Gallery, day 29
Gallery, day 30
Gallery, day 31
Gallery, day 32
Gallery, day 33
Gallery, day 34
Gallery, day 35
Gallery, day 36
Gallery, day 37
Gallery, day 38
Gallery, day 39
Gallery, day 40
Gallery, day 41
Gallery, day 42
Gallery, day 43
Gallery, day 44
Gallery, day 45
Gallery, day 46
Gallery, day 47
Gallery, day 48
Gallery, day 49
Gallery, day 50
Gallery, day 51
Gallery, day 52
Gallery, day 53
Gallery, day 54
Gallery, day 55
Gallery, day 56
Gallery, day 57
Gallery, day 58
Gallery, day 59
Gallery, day 60
Gallery, day 61
Gallery, day 62
Gallery, day 63
Gallery, day 64
Gallery, day 65
Gallery, day 66
Gallery, day 67
Gallery, day 68
Gallery, day 69
delta
lambda

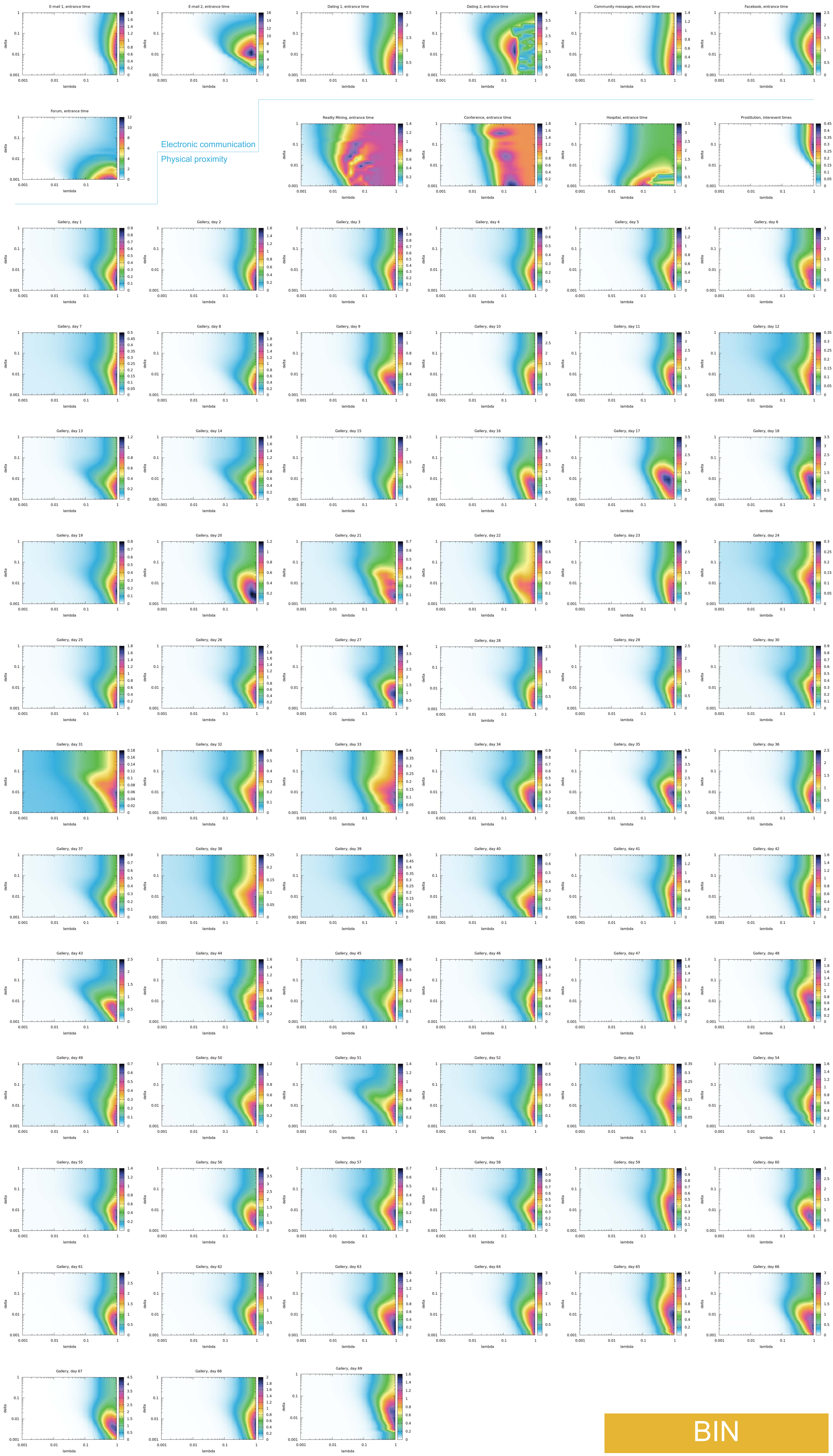

E-mail 1, entrance time
E-mail 2, entrance time
Dating 1, entrance time
Dating 2, entrance time
Community messages, entrance time
Facebook, entrance time
Forum, entrance time
Electronic communication
Physical proximity
Reality Mining, entrance time
Conference, entrance time
Hospital, entrance time
Prostitution, interevent times
Gallery, day 1
Gallery, day 2
Gallery, day 3
Gallery, day 4
Gallery, day 5
Gallery, day 6
Gallery, day 7
Gallery, day 8
Gallery, day 9
Gallery, day 10
Gallery, day 11
Gallery, day 12
Gallery, day 13
Gallery, day 14
Gallery, day 15
Gallery, day 16
Gallery, day 17
Gallery, day 18
Gallery, day 19
Gallery, day 20
Gallery, day 21
Gallery, day 22
Gallery, day 23
Gallery, day 24
Gallery, day 25
Gallery, day 26
Gallery, day 27
Gallery, day 28
Gallery, day 29
Gallery, day 30
Gallery, day 31
Gallery, day 32
Gallery, day 33
Gallery, day 34
Gallery, day 35
Gallery, day 36
Gallery, day 37
Gallery, day 38
Gallery, day 39
Gallery, day 40
Gallery, day 41
Gallery, day 42
Gallery, day 43
Gallery, day 44
Gallery, day 45
Gallery, day 46
Gallery, day 47
Gallery, day 48
Gallery, day 49
Gallery, day 50
Gallery, day 51
Gallery, day 52
Gallery, day 53
Gallery, day 54
Gallery, day 55
Gallery, day 56
Gallery, day 57
Gallery, day 58
Gallery, day 59
Gallery, day 60
Gallery, day 61
Gallery, day 62
Gallery, day 63
Gallery, day 64
Gallery, day 65
Gallery, day 66
Gallery, day 67
Gallery, day 68
Gallery, day 69
lambda
delta
BIN

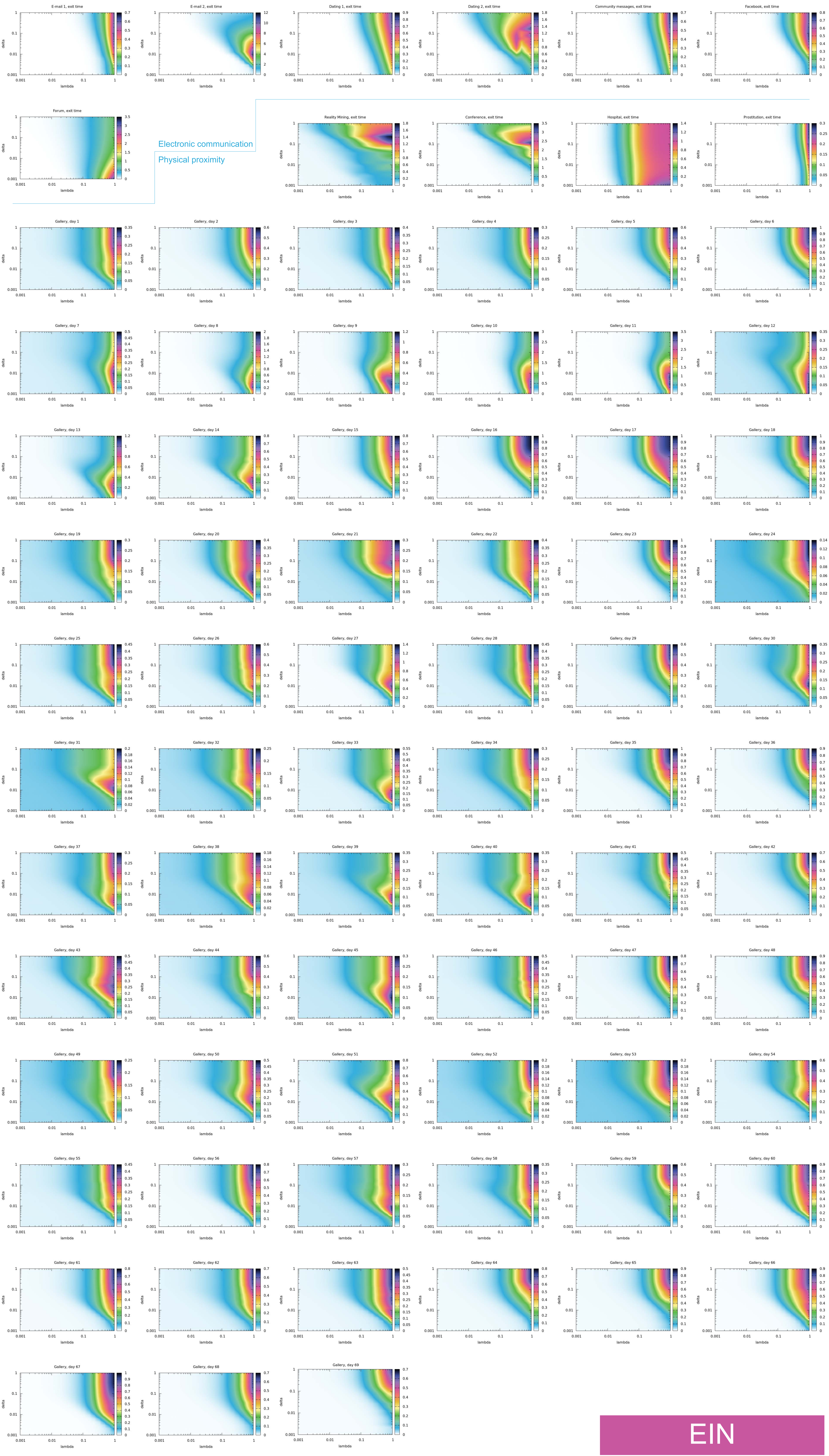

E-mail 1, exit time
E-mail 2, exit time
Dating 1, exit time
Dating 2, exit time
Community messages, exit time
Facebook, exit time
Forum, exit time
Electronic communication
Physical proximity
Reality Mining, exit time
Conference, exit time
Hospital, exit time
Prostitution, exit time
Gallery, day 1
Gallery, day 2
Gallery, day 3
Gallery, day 4
Gallery, day 5
Gallery, day 6
Gallery, day 7
Gallery, day 8
Gallery, day 9
Gallery, day 10
Gallery, day 11
Gallery, day 12
Gallery, day 13
Gallery, day 14
Gallery, day 15
Gallery, day 16
Gallery, day 17
Gallery, day 18
Gallery, day 19
Gallery, day 20
Gallery, day 21
Gallery, day 22
Gallery, day 23
Gallery, day 24
Gallery, day 25
Gallery, day 26
Gallery, day 27
Gallery, day 28
Gallery, day 29
Gallery, day 30
Gallery, day 31
Gallery, day 32
Gallery, day 33
Gallery, day 34
Gallery, day 35
Gallery, day 36
Gallery, day 37
Gallery, day 38
Gallery, day 39
Gallery, day 40
Gallery, day 41
Gallery, day 42
Gallery, day 43
Gallery, day 44
Gallery, day 45
Gallery, day 46
Gallery, day 47
Gallery, day 48
Gallery, day 49
Gallery, day 50
Gallery, day 51
Gallery, day 52
Gallery, day 53
Gallery, day 54
Gallery, day 55
Gallery, day 56
Gallery, day 57
Gallery, day 58
Gallery, day 59
Gallery, day 60
Gallery, day 61
Gallery, day 62
Gallery, day 63
Gallery, day 64
Gallery, day 65
Gallery, day 66
Gallery, day 67
Gallery, day 68
Gallery, day 69
lambda
delta

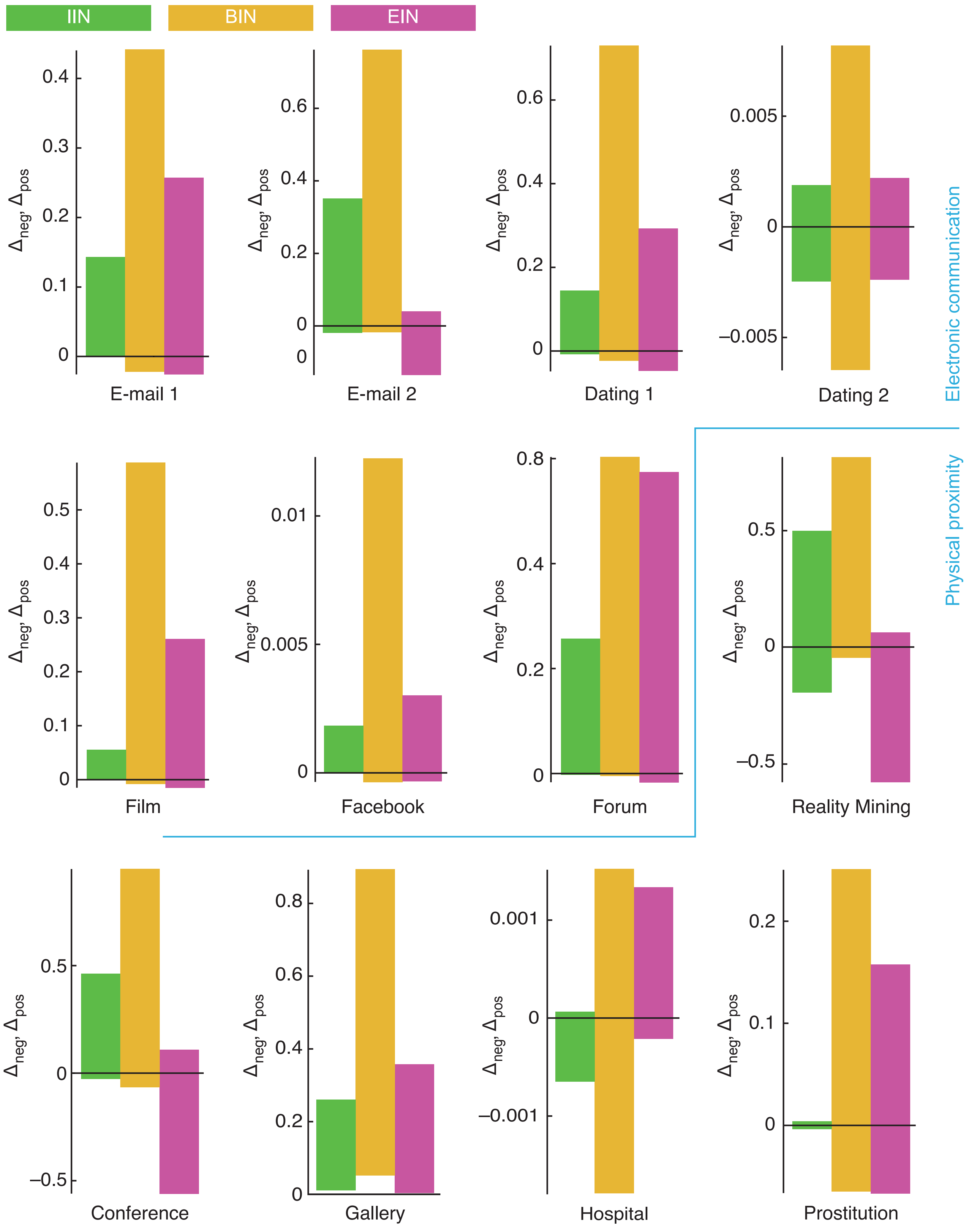

Supporting information 5, maximum deviations for the SIS model

Petter Holme, Fredrik Liljeros

Beginning and end times of relationships control disease spreading in empirical contact networks
